# Overexpression of NOTCH-regulated Ankyrin Repeat Protein is associated with papillary thyroid carcinoma progression

**DOI:** 10.1371/journal.pone.0167782

**Published:** 2017-02-16

**Authors:** Mingdi Zhang, Yiyu Qin, Bin Zuo, Wei Gong, Shenglai Zhang, Yurong Gong, Zhiwei Quan, Bingfeng Chu

**Affiliations:** 1 Department of General Surgery, Xinhua Hospital, Shanghai Jiaotong University, School of Medicine, Shanghai, China; 2 Clinical College, Yancheng institute of Health Sciences, Yancheng, Jiangsu, China; 3 Department of Orthopedic Surgery, Xinhua Hospital, Shanghai Jiaotong University, School of Medicine, Shanghai, China; University of South Alabama Mitchell Cancer Institute, UNITED STATES

## Abstract

Papillary thyroid cancer (PTC) is one of the endocrine cancers with high clinical and genetic heterogeneity. NOTCH signaling and its downstream NOTCH-Regulated Ankyrin Repeat Protein (NRARP) have been implicated in oncogenesis of many cancers, but the roles in PTCs are less studied. In this study, we show that NRARP is frequently over-expressed in thyroid carcinoma. The over-activation of NRARP is highly and positively correlated with NOTCH genes. Moreover, we find that the expression of NRARP is highly associated with several epithelial mesenchymal transition (EMT) markers and contributes to poor survival outcomes. Therefore, these results indicate that NRARP is an important clinical biomarker in thyroid carcinoma and it promotes EMT induction as well as the progression of PTCs via NOTCH signaling activation.

## Introduction

Thyroid carcinoma is the most common type of the endocrine cancers with high clinical and genetic heterogeneity. A rapidly increasing incidence has been reported over the past 30 years [1]. The major type of the thyroid cancer is papillary thyroid carcinoma (PTC), a well-differentiated thyroid cancer derived from follicular thyroid cells. More than 80% of all thyroid cancers are PTCs, which are usually curable with a high five-year survival rate [2]. However, some aggressive and metastatic thyroid cancers can result from poorly differentiated tumor cells. Previous studies reported that activation of mitogen-activated protein kinase (MAPK) signaling pathway is frequently observed in PTC patients. The genetic alterations like BRAF or RAS mutation [3] and RET or NTRK1 fusions [4, 5] can result in the activation of MAPK signaling pathway, and thus lead to dys-regulation of cell differentiation, cell proliferation and cell survival [6]. A recent study reported that NOTCH signaling, another cancer related pathway involved in oncogenic transformation [7, 8], is induced by MAPK signaling and promotes tumor growth in PTC [9].

As the downstream effector of Notch signaling, NOTCH-Regulated Ankyrin Repeat Protein (NRARP) that can be activated by NOTCH pathway was previously reported to be over-expressed in some cancers like breast cancer and liver cancer [10, 11]. In addition, NRARP can act as a mediator to regulate Notch and Lef1-dependent Wnt signaling [12]. NRARP is also involved in liver cancer cell stemness. Depletion of NRARP impairs the stemness of the liver tumor cells and blocked WNT involved cell proliferation [10, 11].

In this study, we hypothesize that NRARP is activated in PTC and induces the cancer progression. This hypothesis motivates us to analyze the TCGA THCA (thyroid carcinomas) dataset [13] and another independent expression data of PTC. Both data show that NRARP expression is significantly up-regulated in PTC. Further bioinformatics analysis has shown that NRARP can interact with NOTCH signaling pathway to induce EMT and lead to poor clinical outcomes. Our analysis demonstrates NRARP expression profiles are clinical relevant and will lead to improved management of patients as well as the personalized treatment.

## Methods and materials

### Gene expression profiling data sets

We used two different expression datasets. One was TCGA THCA (Thyroid carcinoma) dataset containing around 500 tumor samples and 60 normal samples, of which nearly all the cancer samples are papillary thyroid carcinomas (PTCs)[13]. The other was GSE64912: RNA-Sequencing of human PTCs [14], which contains 4 healthy donors' thyroids and 18 PTCs. TCGA data (normalized log2 RSEM files) were downloaded from FireBrowser database (http://firebrowse.org). The public GSE64912 dataset was obtained from NCBI GEO database (http://www.ncbi.nlm.nih.gov/geo/), which is the raw count file. The data sets are shown in the supporting information files.

### Differential expression analysis and correlation analysis

In TCGA data, we applied nonparametric test: Wilcox rank test to identify the differential gene based on the normalized RSEM values. In GSE64912 data, we applied Deseq2 to call the differential genes based on raw read counts. Pearson correlation coefficients were used to measure the expression correlation.

### Pathway enrichment analysis

GSEA software [15] was performed to identify enriched pathways. KEGG pathway database was chosen to annotate genes. Up-regulated pathway enrichment was calculated by using gene fold changes. Positively correlated pathway enrichment was calculated by using correlation coefficients with NRARP.

### Survival analysis

The statistical method used to explore the relationship between the survival of a patient and mutation variables is log rank test which was used to calculate the relationship significance by using the package “Survival” in R [16] (version 3.2.3). Kaplan Meier plot was also generated through this package.

### Protein protein interaction analysis

Protein protein interactions information was gained from database. Official gene symbols were uploaded and the organism selected is Homo-sapiens.

## Results

### NRARP is frequently over-activated in thyroid carcinoma

**Fig 1:**
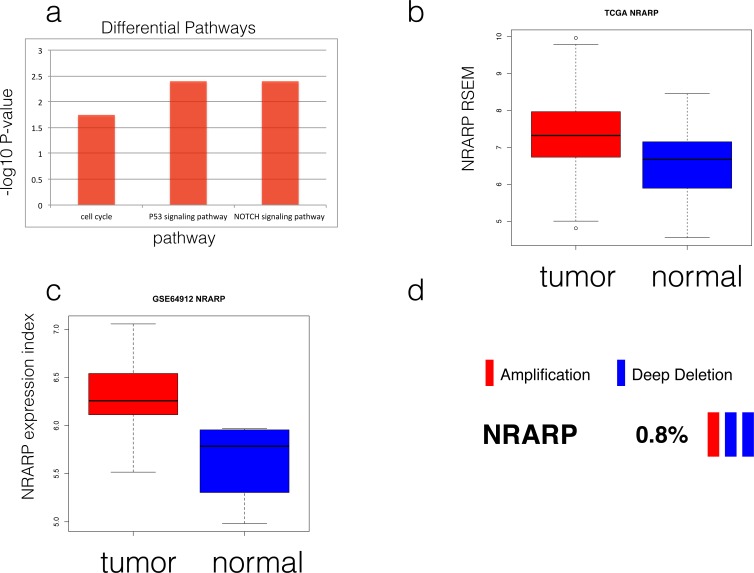
Over-expression of NRARP in thyroid tumors. A) Up-regulated pathways in TCGA THCA dataset. B) Boxplot of NRARP expression profiles in TCGA THCA dataset. C) Boxplot of NRARP expression profiles in public dataset GSE64912. D) Genomic alteration frequency of NRARP in TCGA THCA dataset. Each cell stands for the tumor patient.

To identify the cancer related pathways and oncogenes in thyroid carcinoma, we analyzed the TCGA THCA data containing more than 500 samples. We found up-regulated genes are significantly enriched in NOTCH signaling pathway (p = 0.018), cell cycle (p = 0.004), P53 signaling pathway (p = 0.004) (Fig 1A). These significant pathways are consistent with the previous study [9]. Recently, Dongwon Choi et al. discovered that NOTCH signaling is aberrantly increased in PTC and promotes the malignant phenotypes of thyroid carcinoma cells [17]. These findings highlighted the tumor promotion functions of NOTCH activation in malignant thyroid tumors. As expected, NRARP, a Notch related mediator and downstream [11] is also up-regulated in tumor patients when compared with the normal samples. Of Note, up-regulation is reproducible in another public dataset (Log2 fold change = 0.80, P = 1.347e-08 and log2 fold change = 0.95, p = 0.0013(Fig 1B and 1C)). The similar expression pattern implied that NRARP is frequently activated in mRNA level and may play an oncogenic role in thyroid cancer. Nevertheless, the alterations of copy number or somatic mutations are unlikely to occur in thyroid carcinoma. No somatic mutations were observed and the copy number variation (CNV) frequency is quite low. Only 0.88% patients gain CNVs based on the cBiopotal database (amplication: 1/399 and deletion: 2/399, respectively, Fig 1D). These results suggested that NRARP is activated in THCA only in expression regulation levels rather than in genomic DNA levels.

### NRARP over-activation associated with NOTCH signaling pathways and cancer progression in thyroid carcinoma

**Fig 2:**
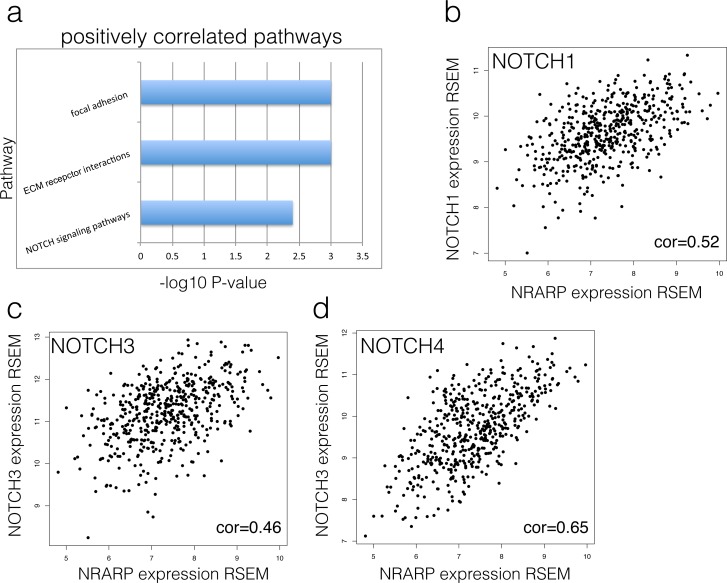
Expression correlation between NRARP and NOTCH signaling. A) Enriched pathways that are positively correlated with NRARP. B) Scatter plot of NRARP expression and NOTCH1 expression in TCGA THCA dataset. Each point stands for one tumor patient, of which values in X-axis and Y-axis represent NOTCH1 and NRARP expression, respectively. C) Scatter plots of NRARP and NOTCH3 genes. D) Scatter plots of NRARP and NOTCH4. E) Protein interaction between NRARP and NOTCH genes.

To investigate the NRARP functions of tumorigenesis in tumor, we further performed a correlation analysis in TCGA tumor samples. We found NRARP positively correlated genes are significantly enriched in NOTCH signaling pathways (P = 0.004), ECM receptor interactions (P<0.001) and focal adhesion (P<0.001) (Fig 2A). Notably, NRARP gene showed significant positive correlation with NOTCH genes like NOTCH1, NOTCH3 and NOTCH4 (Fig 2B–2D). All the three NOTCH genes as well as NRARP are up-regulated in tumors (S1 Fig). Although NOTCH1 up-regulation in GSE64912 is not as significance as that in TCGA due to the small sample size, the trend is similar. Both datasets displayed increased expression profiles of all three NOTCH genes in tumors compared with the normal samples (S1 Fig). The expression consistency of NRARP and NOTCH genes occurring not only within the tumor patients also between the tumors and normals indicated a strong regulation relationship in PTCs. In addition, Protein interactions enhanced this regulation relationship between NRARP and NOTCH genes. Besides, pathway analysis also indicated that the patients with higher NRARP expression accompany dysregulation of Focal adhesion and ECM receptor interactions. These two pathways are closely related to cancer invasion and metastasis [18, 19]. These observations strongly revealed that the over-activation of NRARP exerted important impacts on thyroid carcinoma progression in a NOTCH related manner.

### EMT induction is concomitant with NRARP increasing in thyroid cancer

**Fig 3:**
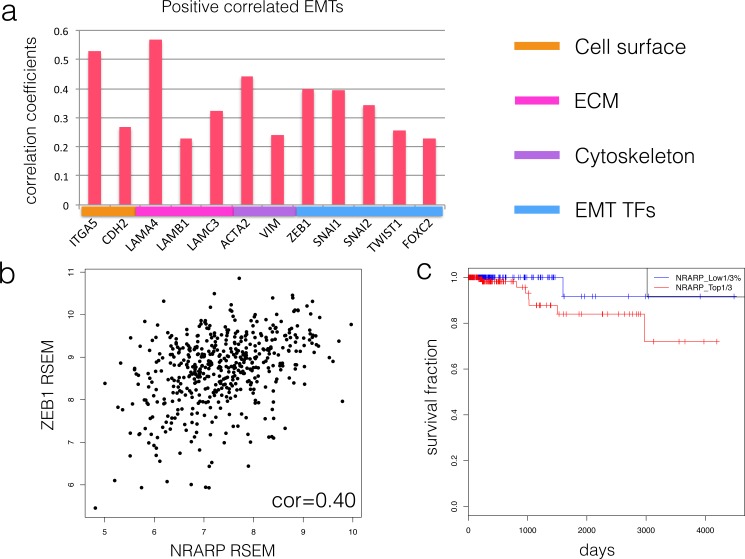
NRARP as an outcome predictor for thyroid carcinoma patients. A) Correlation coefficients between NRARP and the EMT markers that are positive correlated with NRARP in TCGA THCA dataset. Each gene is annotated and labeled according to its function in EMT. B) Scatter plot of NRARP expression and ZEB1 expression in TCGA data. C) Kaplan Meier survival curves of patients with the highest 1/3 of the NRARP expression (red) and patients with the lowest 1/3 of the NRARP expression (blue).

We have identified NRARP as an oncogene candidate over-expressed in THCA and its over-activation can contribute to thyroid tumor aggression by correlating with NOTCH, but the mechanisms are less clear. To understand the molecular mechanisms of NRARP involved in thyroid cancer progression, we further detected the linkage between EMT and NRARP. Epithelial mesenchymal transition (EMT) is a vital pathologic mechanism in most tumor progression including thyroid cancer [20]. Association analysis revealed that higher expression correlates with higher activation of several EMT markers in tumors (Fig 3A and 3B, S1 Table). ZEB1, N-cadherin(CDH2), SNAI1, SNAI2, TWIST1, FOXC2 and laminin genes like LAMA4, LAMB1, LAMC3 [21] are the most significant EMT markers that are positively correlated with NRARP expression (Fig 3A and 3B, S1 Table). Furthermore, most pairwise expression patterns are reproducible or similar in another independent dataset except for a few factors. The little inconsistency probably results from the smaller sample size (22 samples in all) that may cause more noise and poor accuracy. These positively correlated genes are known to be acquired during the transition whose functions are involved in transcription regulatory, cell surface or ECM (extracellular matrix) [21]. The experiment by Zhu Zusen group proved that NRARP knockout led to attenuate cancer cell stemness [10] which is linked with EMT by TWIST1 and ZEB1, two EMT related transcription factors that are also highly correlated with NRARP [22, 23]. Besides, the recent study which revealed that increased NRARP is involved in NOTCH-induced EMT in colon cancer also enhanced the functional association between NRARP and EMT [24]. Our correlation analysis and literature mining suggested that NRARP promotes EMT and cancer invasion via NOTCH signaling activation.

### NRARP expression is a clinical biomarker in thyroid carcinoma

NRARP is frequently over-expressed in thyroid cancer whose activation may contribute to EMT depending on activated NOTCH signaling pathways. Although thyroid carcinomas have a better overall survival rate than other malignancies, in occasional cases they can still dedifferentiate into more aggressive and lethal thyroid cancer [13]. Therefore, clinical survival predictors are still useful for providing better care for the patients. Consistent with the previous studies [2], the overall risk of death from thyroid cancer is about 5%. However, compared to the patients with lowest NRARP expression (bottom 33.3%), those whose NRARP expression is highest (top 33.3%) have a worse survival rate (Log rank p = 0.04558, Fig 3C). The results demonstrated that NRARP expression is a predictive biomarker for the outcome of the thyroid cancer.

## Discussion

NRAPR as well as the NOTCH pathway has been implicated in the pathogenesis of different types of cancer respectively. However, how NRARP aggravate the cancer was less known. In this study, we uncovered NRARP expression is increased in PTC, which are associated with NOTCH pathway activation. Further correlation analysis revealed that NRARP expression is positively correlated with some vital EMT markers that are acquired during the transition. These results are reproducible in a different dataset, which point out that our findings are general in the thyroid cancer progression rather than specific to a single-data set. We also discover NRARP up-regulation is the predictor of poor outcome. Together, these observations all support our hypothesis that NRARP works as the oncogene contributing the thyroid tumor aggression.

NOTCH pathway can induce NRAPR expression, as is the downstream effectors of the NOTCH signaling. However, several studies also reported NRARP can act as a mediator to regulator NOTCH signaling [12, 25]. The regulatory relationship between NOTCH and NRAPR is complicated and tight [10, 12]. Here we reveal that NRARP can coordinate with NOTCH genes to regulate thyroid cancer aggression and to stimulate EMT. NRARP and some other genes involved in NOTCH pathways are all over-expressed in thyroid cancer. Besides, NRARP expression is also positively correlated with such NOTCH related genes across the tumor patients by excluding normal samples. Noteworthy, NRARP silencing can reduce the breast tumor proliferation, which proves NRARP itself can work as a driver oncogene instead of a passenger, of which over-activation promotes tumor cell growth or survival [11]. These experiments[11] at least partially support our findings that NRARP plays a direct oncogenic role in connecting NOTCH signals to thyroid cancer progression and disprove it is only the ‘random product’ of cancer development and carry no functional significance. These published results help complement the weakness that expression correlation itself is not able to directly prove the causality [26]. Finally, we tried another independent dataset and confirmed that our findings are reliable in thyroid carcinoma.

**Fig 4:**
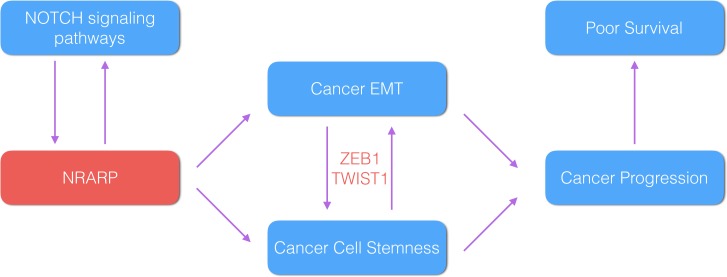
Proposed model regulatory model of NRARP in thyroid cancer patients. NRARP and NOTCH related genes are up-regulated in tumor patients and contribute to tumor progression. See discussion for details.

Based on these literature evidences and our analysis results, we propose a regulatory model of NRARP activated in PTC pathogenesis. NRARP is increased by NOTCH signaling and in turn regulate NOTCH signaling as well as cooperate with it to induce cancer EMT and cancer stemness. At last EMT facilitates cancer progression and lead to a worse clinical outcome (Fig 4).

In conclusion, our work establishes a strong linkage between NRARP and thyroid carcinoma progression. NRARP dysregulation can contribute to aggression and lethality of the thyroid cancer. Since NRARP participates in thyroid malignancies development and correlates with poor survival, our analysis provides a novel insight into the prognostic marker for thyroid cancer. Future comprehensive identification of therapeutic biomarkers holds the key to personalized medicine for the better treatment of thyroid cancer patents.

## Supporting information

S1 Fig Expression of all three NOTCH genes in tumor and normal samples. (TIF)

S1 File data set from TCGA. (TXT)

S2 File data set from TCGA. (TXT)

S3 File data set from TCGA. (RAR)

S1 Table Correlation coefficients between NRARP genes and related genes in TCGA dataset and another independent dataset, respectively. The First column is the annotated function of the NRARP partner gene. The second and the third column are partner genes and NRARP. The last two columns show the correlation coefficients in TCGA data and GSE64912.
(XLSX)
