# Supplementary figures and images for: Overexpression of NOTCH-regulated Ankyrin Repeat Protein is associated with papillary thyroid carcinoma progression
Source: PLoS One. 2017 Feb 16;12(2):e0167782. doi: 10.1371/journal.pone.0167782 (PMC5312965; doi:10.1371/journal.pone.0167782)

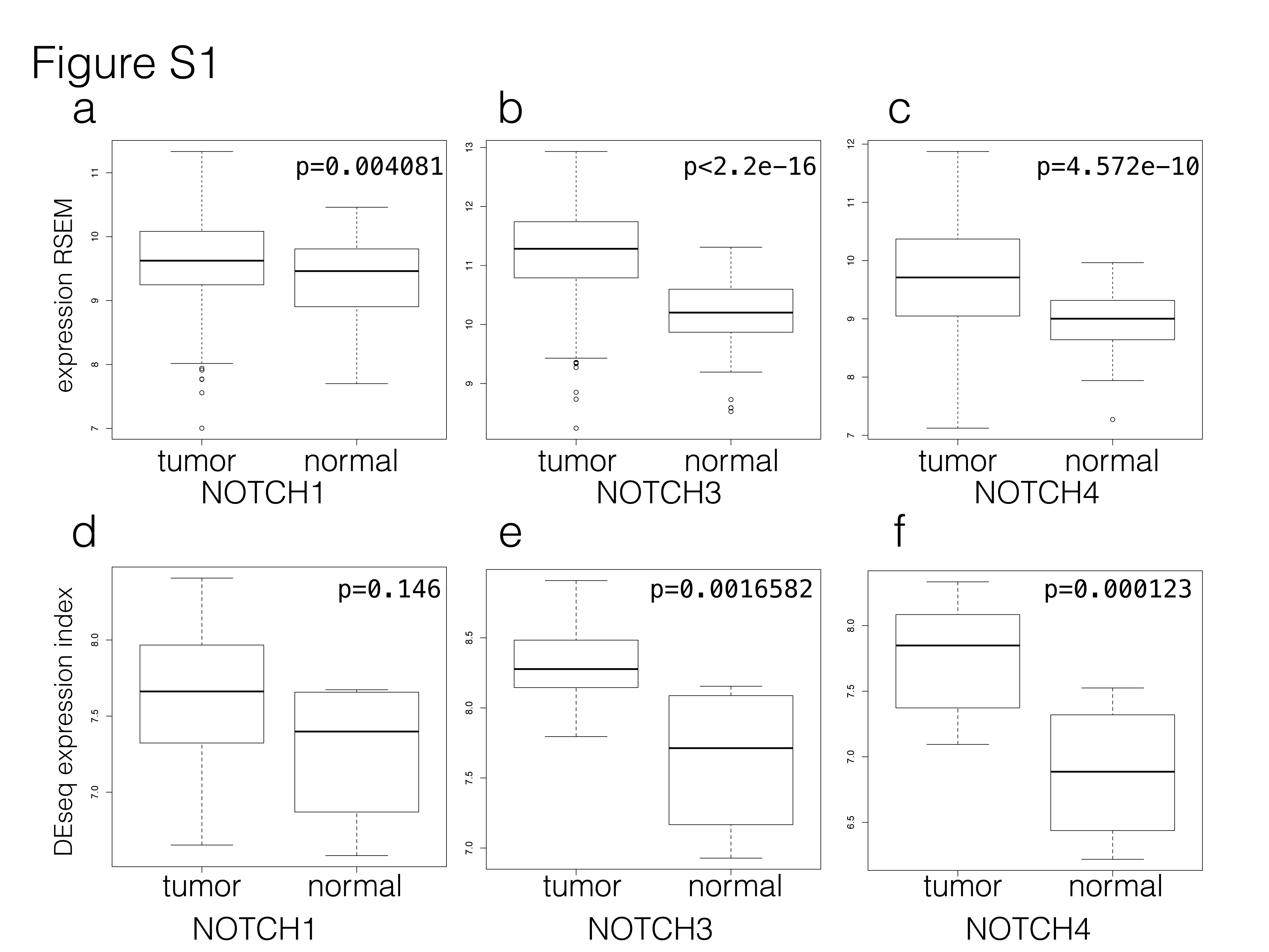

Supplement: S1 Fig — (TIF) [file pone.0167782.s001.tif]
